# Supplementary material for: Content-rich biological network constructed by mining PubMed abstracts
Source: BMC Bioinformatics. 2004 Oct 8;5:147. doi: 10.1186/1471-2105-5-147 (PMC528731; doi:10.1186/1471-2105-5-147)
Supplement: Additional File 2 — The original results of the above study (non-essential files are deleted to keep the file size under the limit set by BMC bioinformatics). [file 1471-2105-5-147-S2.bz2 › chilibotAdditionalFile2/dip05/418ID8702914E10287/html/BMPR1B_GDF5.html]

 


 **BMPR1B** and **GDF5** 
  
Found 4 abstracts in PubMed, retrieved 4.  
 

 What does Google say? 
 PDF only 
| .edu only 

---

**Interactive relationship** (e.g. stimulation, inhibition, etc)

**Neutral relationship**- GDF 5  [ **GDF5** ]  bound to ALK 6  [ **BMPR1B** ]  BMPR IB and BMP type II receptor BMPR II but not to ALK 3 BMPR IA in ROB C26 cells.  Ref: 9125848 Kokubyo Gakkai Zasshi, 1997
- Analysis using COS 1 cells revealed that GDF 5  [ **GDF5** ]  bound to ALK 6  [ **BMPR1B** ]  BMPR IB, but not to the other type I receptors when expressed alone.  Ref: 9125848 Kokubyo Gakkai Zasshi, 1997
- Moreover, GDF 5  [ **GDF5** ]  transduced the signal efficiently by ALK 6  [ **BMPR1B** ]  BMPR IB in the presence of BMPR II or ActR II.  Ref: 9125848 Kokubyo Gakkai Zasshi, 1997
- BMP 4 and GDF 5  [ **GDF5** ]  are known to bind to activin receptor like kinase 3 ALK 3 and or ALK 6  [ **BMPR1B** ]  also termed BMP type IA and type IB receptors, respectively.  Ref: 11282024 J Cell Sci, 2001

**Non-interactive relationship** (e.g. studied together, co-existance, homology, etc.)

- In the presence of type II receptors, GDF 5  [ **GDF5** ]  bound to different sets of type I receptors, but the binding was most efficient to ALK 6  [ **BMPR1B** ]  BMPR IB compared to the other type I receptors.  Ref: 9125848 Kokubyo Gakkai Zasshi, 1997
